# Consensus on core elements and methodological approaches for research on non‐pharmacological intervention in dementia: Protocol for a Delphi study

**DOI:** 10.1002/alz70858_102714

**Published:** 2025-12-26

**Authors:** Sara Laureen Bartels, Federica D'Andrea, Maud Graff, the INTERDEM Methodology Taskforce

**Affiliations:** ^1^ Maastricht University, Maastricht, Limburg, Netherlands; ^2^ The Geller Institute of Ageing and Memory, University of West London, London, London, United Kingdom; ^3^ Department of IQ Health, Radboudumc Alzheimer Center, Radboud University Medical Center Nijmegen, Nijmegen, Gelderland, Netherlands; ^4^ Department of Rehabilitation, Radboudumc, Radboud University Medical Centre Nijmegen, Nijmegen, Gelderland, Netherlands

## Abstract

**Background:**

The six core elements of the Medical Research Council (MRC) framework for developing, evaluating and implementing complex interventions include 1) consider context, 2) develop, refine, and (retest) program theory, 3) engage stakeholders, 4) identify key uncertainties, 5) refine interventions, and 6) economic considerations (Skivington et al., 2021). It is currently unclear if these elements are perceived as relevant, if additional elements should be considered, and which methodological approaches best operationalize them when developing, evaluating and implementing non‐pharmacological interventions for dementia.

This Delphi study is part of the METHODEM project and aims at i) determining which core elements are crucial for non‐pharmacological interventions in dementia care and ii) reaching a consensus on which methodological approaches can best integrate these core elements and address the objectives in the development, evaluation and implementation of research on non‐pharmacological interventions for dementia.

**Method:**

A stakeholder‐informed Delphi design will be used. In round 1, international, multi‐disciplinary researchers will be asked to rate the importance of the MRC core elements per phase (i.e., development, feasibility/piloting, evaluation, implementation), to suggest additional core elements, and list methods/methodologies that best consider core elements. The round 1 results will be discussed in public involvement events with stakeholders (e.g., people with lived experiences, policy makers, care professionals) for a comprehensive perspective. In round 2, the list of core elements and methods/methodologies, and stakeholders’ perspectives will be fed back to researchers, resulting in rankings. This process will be repeated until a consensus on core elements and methodological approach is achieved.

**Result:**

The first round of the Delphi study is expected to be completed by the summer 2025.

**Conclusion:**

To advance the field of non‐pharmacological research in dementia, methodological rigor is essential. Central in how research is done is also ensuring that academic processes are feasible and relevant for all stakeholders. Traditional approaches such as Randomized Controlled Trials may not answer the question what works for whom how why, thus, a consensus on alterative methods and methodologies is needed to guide future research.

